# Tobacco industry data on illicit tobacco trade: a systematic review of existing assessments

**DOI:** 10.1136/tobaccocontrol-2018-054295

**Published:** 2018-08-22

**Authors:** Allen W A Gallagher, Karen A Evans-Reeves, Jenny L Hatchard, Anna B Gilmore

**Affiliations:** Tobacco Control Research Group, University of Bath and UK Centre of Tobacco and Alcohol Studes, Bath, UK

**Keywords:** illegal tobacco products, tobacco industry, public policy

## Abstract

**Objective:**

To examine the quality of tobacco industry-funded data on the illicit tobacco trade (ITT) through a systematic review of existing assessments of industry-funded data on ITT.

**Data sources:**

Papers and reports assessing tobacco industry-funded data on ITT were obtained via searches of 8 academic databases, Google searches and correspondence with ITT experts.

**Study selection:**

Inclusion criteria identified 35 English-language papers containing an original assessment of tobacco industry-funded data.

**Data extraction:**

Using a coding framework, information was extracted from the assessments regarding the quality of tobacco industry data. Documents were second-coded, achieving 94% intercoder reliability with all disagreements resolved.

**Data synthesis:**

Of the 35 assessments reviewed, 31 argued that tobacco industry estimates were higher than independent estimates. Criticisms identified problems with data collection (29), analytical methods (22) and presentation of results (21), which resulted in inflated ITT estimates or data on ITT that were presented in a misleading manner. Lack of transparency from data collection right through to presentation of findings was a key issue with insufficient information to allow replication of the findings frequently cited.

**Conclusions:**

Tobacco industry data on ITT are not reliable. At present, the tobacco industry continues to fund and disseminate ITT research through initiatives such as PMI IMPACT. If industry data on ITT cannot meet the standards of accuracy and transparency set by high-quality research publications, a solution may be to tax tobacco companies and administer the resulting funds to experts, independent of the tobacco industry, who use previously developed reliable models for measuring ITT.

## Introduction

The illicit tobacco trade (ITT) is difficult to measure due to its illegality,^1–3^ its global^4^ and changing nature^2 5^ and data collection and analysis complexities.^2 3 6 7^ While methods such as Empty Pack Surveys (EPS), consumer surveys, econometric modelling and tax gap approaches have been used effectively by multiple non-industry sources;^3 8–15^globally, there is no agreed ‘gold-standard’ methodology for estimating ITT^16^ and estimates vary greatly in rigour and approach.^17^

In recent years, transnational tobacco companies (TTCs) (box 1) have been a major funding source of data on ITT.^18–28^ They heavily publicise these data, especially when a tobacco control policy is being debated.^1^ The tobacco industry has commissioned reports on ITT,^19 25–27 29–35^ often produced by global accountancy firms such as KPMG, Deloitte and PricewaterhouseCoopers.^25 28 32^ At least one of these firms has expressed concern that the TTCs have used their research findings in a misleading manner.^36^ TTCs use such self-funded data and the threat of the ITT in efforts to oppose tobacco control policies,^1 37 38^ arguing that tobacco control measures will increase ITT and its associated criminality.^1 37–44^

Box 1 **Important terminology**

**Transnational tobacco company/companies**

Transnational tobacco company/companies (TTCs), the major four currently being British American Tobacco,^105^ Imperial Tobacco,^106^ Japan Tobacco International^107^ and Philip Morris International.^108^

**Counterfeit**

Products bearing a trademark of a cigarette manufacturer that are manufactured by a third party without the cigarette manufacturer’s consent.^46^

**Tobacco industry illicit**

Tobacco company product that was en route to, imported into, distributed in or sold in a jurisdiction in violation of the applicable fiscal laws of that jurisdiction,^46^ for example, tobacco industry product present in the illicit market. The fact that this product was manufactured by the TTCs does not imply they are always responsible when that product ends up on the illicit market.

**Contraband**

Any tobacco product (including counterfeit and tobacco industry) imported in a jurisdiction in violation of the applicable fiscal laws of that jurisdiction.^55^

**Non-domestic**

Tobacco products brought in from an overseas market. This can include overseas purchases that were then transported legally (legal non-domestic), as well as contraband products (illicit/illegal non-domestic).^109^

As a consequence of TTCs' use of self-funded data, a growing number of independent studies have scrutinised the quality of these data in Australia,^45^ Europe,^46^ Asia^47^ and South Africa,^48^ levelling a number of criticisms against them.^31 46^ To date, there has been no attempt to systematically summarise this literature. Undertaking these assessments is expensive, time consuming and difficult to achieve quickly enough to be useful within the rapidly moving policy cycle.

This paper therefore aims to systematically review existing studies, which assess tobacco industry data on ITT (hereafter ‘assessments’) to provide a substantive overview of the characteristics of such data and to identify the nature of critiques of tobacco industry data/reports on ITT. By compiling this information, this review will aid public health responses to any future data on this topic.

Growing tobacco industry funding of research on ITT underlines the importance of such work. Philip Morris International’s (PMI) latest initiative, PMI IMPACT, has pledged US$100 million to fund research on illegal trade and related crimes and, as of early 2017, had committed US$28 million to 32 projects across the European Union (EU).^49 50^ Outlining the findings of existing assessments of past data funded by the tobacco industry is a useful and necessary step towards better understanding future data and how to respond to it.

## Methods

### Search

To identify existing independent assessments of TTC-funded data or reports on the ITT, the following search string was applied to eight databases (Business Source Complete, Embase, the International Bibliography of Social Sciences, Ovid, PubMed and PubMed Central, ScienceDirect and Web of Science):

(("Philip Morris" OR "PMI" OR "British American Tobacco" OR "BAT" OR "Imperial Tobacco" OR "Imperial Brands" OR "Imperial" OR "ITG" OR "Japan Tobacco" OR "JTI" OR "Tobacco company" OR ‘"transnational tobacco company" OR "TTC" OR "TTCs") AND ("research" OR "evidence" OR data* OR "study" OR "studies" OR report*) AND (illicit* OR illegal* OR smuggl* OR "contraband" OR counterfeit*) AND ("tobacco" OR cigar*)).

Minor variations were made in order to identify the most effective search for each database. Additional searches were conducted in the specialist peer-reviewed journals *Addiction*, *Health Economics* and *Tobacco Control* to ensure that potentially relevant assessments had not been overlooked within the main database searches.

Google searches were performed to identify grey literature using ‘illicit tobacco’ and the names of the aforementioned TTCs. Searches for ‘illicit tobacco’ were also performed on websites of organisations involved in tobacco industry monitoring and research on ITT. All searches were conducted between February and March 2017. In order to capture as many potentially relevant assessments as possible, results were not restricted by year of publication (see online supplementary appendix 1 for full protocol).

10.1136/tobaccocontrol-2018-054295.supp1 Supplementary file 1

A total of 3815 potential assessments were identified; 3720 from database searches and 95 from non-database sources. Records were stored in a reference management system (Endnote) where duplicates were removed, leaving 2690 documents. Inclusion and exclusion criteria (box 2), developed in conjunction with all authors and piloted, were then applied, leaving 56 potential assessments after title and abstract screening. The bibliographies of these 56 were then hand-searched to identify any additional literature, bringing the total of potential assessments to 60.

Box 2 **Inclusion and exclusion criteria and key definitions.**

**Inclusion criteria**

This review aimed to identify documents that assess tobacco industry-funded data on illicit tobacco trade (ITT) (assessments) and was conducted in two stages:

1. Title/abstract screening:
  - Document must be written in English.
  - Document must include data on ITT (a key term search of the document was conducted when this could not be determined from the title or abstract).
2. Full-text screening:
  - Document must not have received funding from the tobacco industry.
  - Document must assess data on ITT that has received funding from the tobacco industry.
  - Document must clearly identify the data that are being assessed, eg, the source of the data has to be identifiable from the contents of the document.

**Key definitions**

*‘Assess’*=to provide an evaluation of tobacco industry data on illicit trade. This could be a positive or negative statement regarding any element of the data such as how it was collected, analysed, presented, etc referring to or citing data without providing any critical comment on it was not considered an assessment of that data. Solely referring to pre-existing critiques of data was also not considered an assessment.

*‘Industry-funded data on illicit trade’*=any data on illicit tobacco that has been funded fully, or in part, by tobacco companies including industry-commissioned research and research conducted by those that receive industry funding. This includes data that transnational tobacco companies claim as their own or have commissioned, as well as data featured in a newspaper, website, public event or advertising campaign that comes from an industry source.

*‘Source’*=where the assessed data were taken from. Sources may include industry-commissioned reports, internal industry documents, industry press releases and media reports containing statements made by tobacco companies or their representatives.

**Figure 1:**
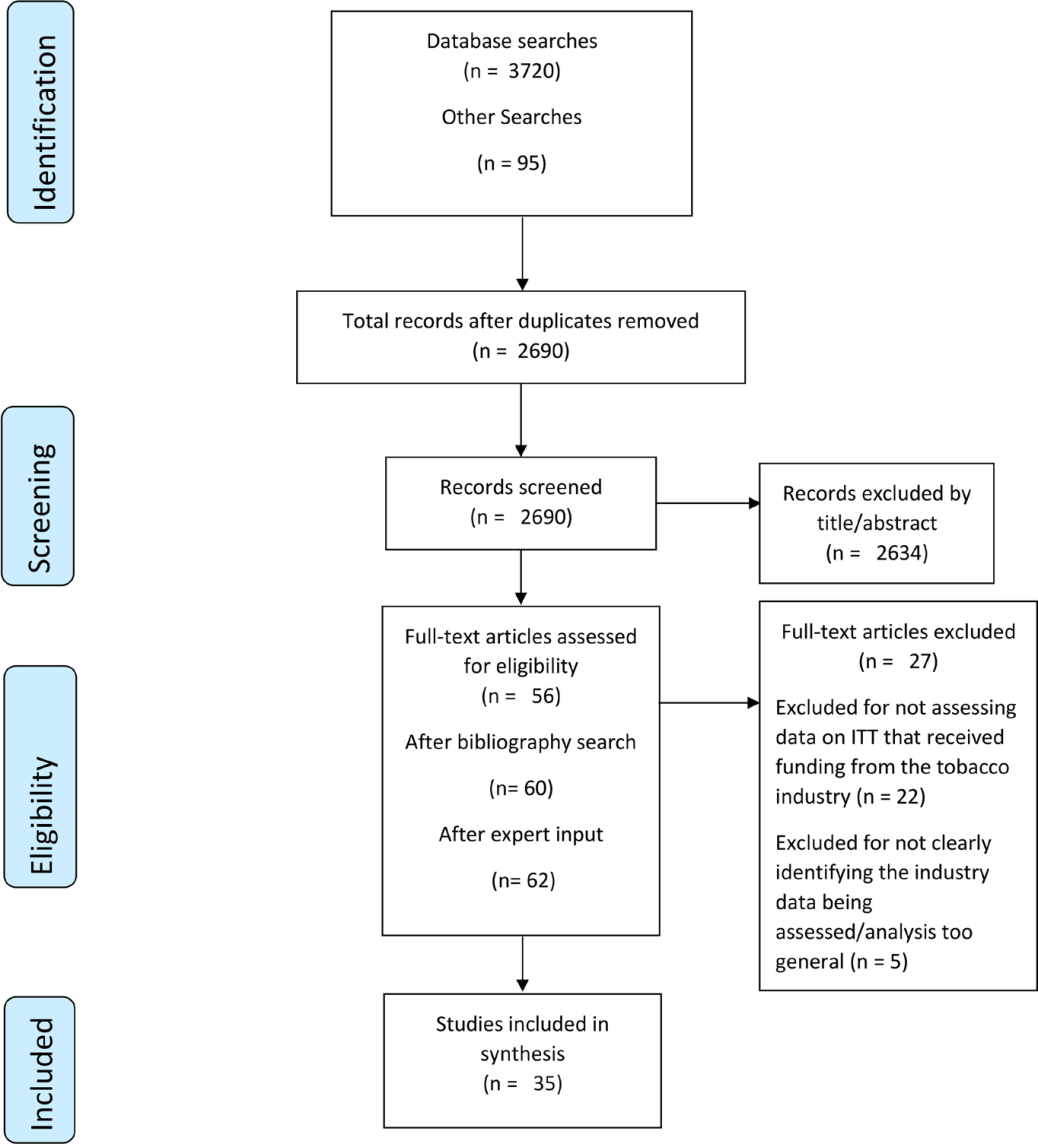
Study selection process—Preferred Reporting Items for Systematic Reviews and Meta-Analyses flow diagram. ITT, illicit tobacco trade.

These 60 were then read in full for relevance, defined as ‘providing an original assessment of industry data on ITT and clearly identifying the source of the data’ (box 2). Tobacco control experts with an interest in ITT were then asked to review a list of assessments that had been deemed eligible after full-text review, and asked for any additional literature to include in the review and any other experts to contact. Experts identified two additional articles. A total of 35 assessments were included in the review (figure 1, online supplementary appendix 2).

### Data extraction and coding

Critical appraisal of the assessments themselves was conducted, considering if they underwent a peer-review process, disclosed funding sources and outlined their methodological approach (see online supplementary appendix 3). Second, using a framework developed by all authors (see online supplementary appendix 4), we coded five key aspects of the industry data (covering characteristics and criticisms/praise of them (table 1)) as detailed in the 35 assessments. This framework, refined after being piloted on a sample of three assessments, was based on existing literature on methods for measuring ITT^3 51–53^ and Ross’ criteria^54^ for assessing the quality of estimates on tobacco tax avoidance and evasion.

**Table 1:** Key categories of industry data (based on assessments) captured by coding framework

Characteristics	Title/year/funder	The organisation that produced the data, the title and year of report (if applicable) and the funder of the data.
	Geographic information	The region, country and locality of the data (each included when applicable).
	Data collection type (can select multiple)	Survey (tobacco consumer). Survey (tobacco retailer). Empty Pack Surveys. Industry sales data. Seizure data. Expert input. Export and import/international trade statistics.
	Analytical method used (can select multiple)	Quantitative analysis. Flows model. Tax gap. Econometric modelling. Qualitative analysis. Comparison of export and import statistics.
Criticisms/praise	Criticisms made (with option to highlight praise)	Estimates were substantially higher than comparable independent estimates. Criticism of data collection methodology. Criticisms of analysis. Poor presentation of results. Funding is a conflict of interest. Author/s do not take responsibility for findings. Not peer-reviewed. Research contributes nothing of value. Funding is not acknowledged.

The critical appraisal and coding processes were recorded in an excel spreadsheet and 100% double-coded, resulting in a mean level of 94% intercoder reliability. All disagreements were documented and fully resolved. As lack of transparency emerged as an over-arching theme within criticisms of industry data, a qualitative overview of transparency-related statements made in assessments (captured using NVivo, a computer software supporting qualitative analyses) was also conducted.

## Results

### Characteristics of the assessments

Our sample of 35 assessments were all published post-2000. Twenty-five (71%)^37 41 44 45 48 55–74^ assessed tobacco industry-funded country-level estimates, five (14%)^46 47 75–77^ assessed region-wide (eg, EU, Asia) estimates and five (14%)^1 54 78–80^ featured assessments at both regional and country-level. Twenty-one (60%)^1 37 41 44–48 54 56 58 59 61 62 64 65 72 73 76 77 80^ outlined their methodological approach, 18 (51%)^37 41 44–48 56 59 61 64 65 67 72–74 76 77^ featured in a peer-reviewed publication and 18 (51%)^1 37 41 44–48 54 56 61 62 64 73 74 76 77 79^ disclosed their funding source/s.

### Characteristics of industry data: geography, data type and analytical method

**Figure 2:**
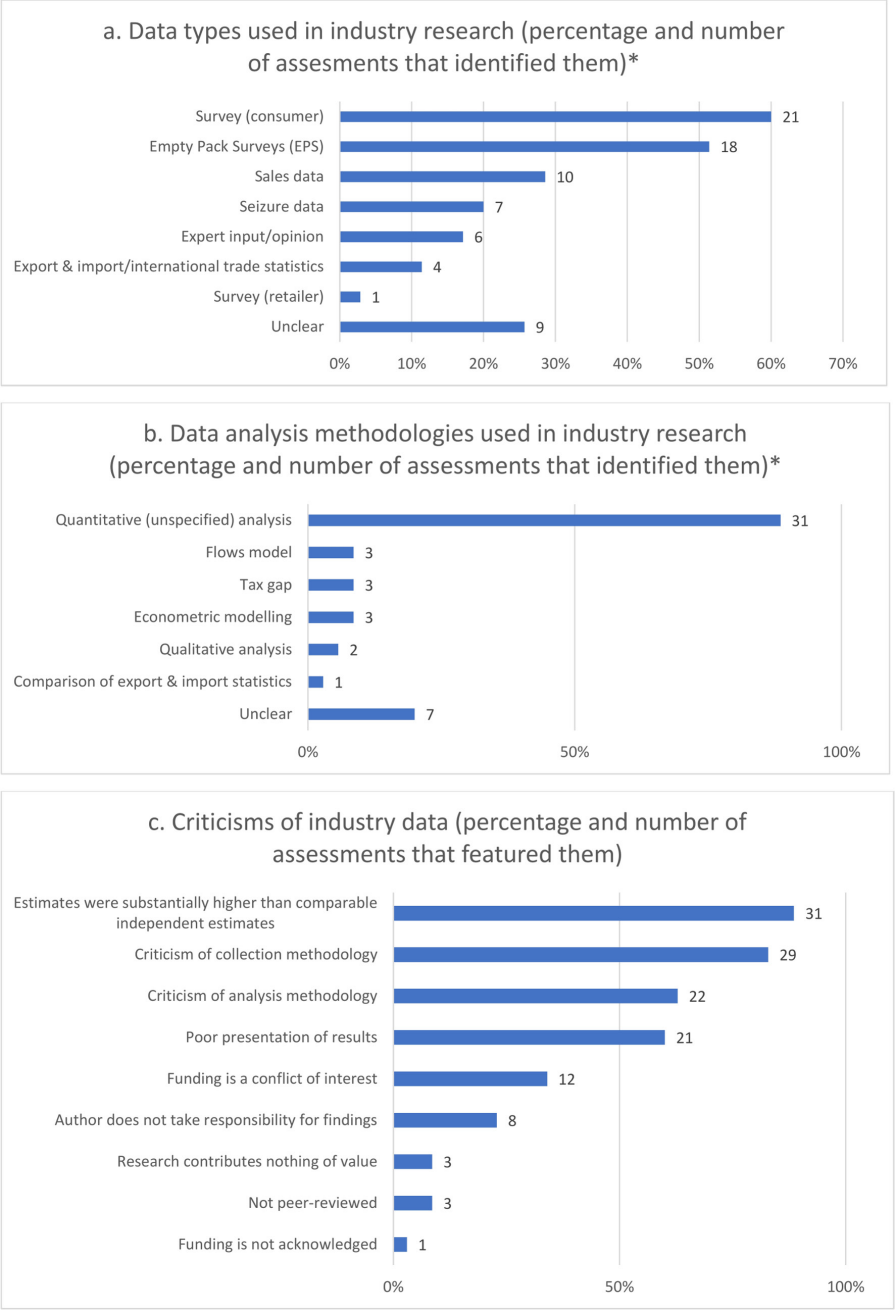
Identified data collection methodologies, analytical methods and criticisms. *Total percentages add up to >100% due to more than one methodology being identified.

The most commonly featured countries in the literature were Australia (37)^1 41 45 54–60 63 65 68–71 78–80^ and the UK (9),^1 37 41 74^ with region-wide data most often relating to the EU (6)^1 46 54 76 77 79^ and Asia (5)^47 54 75 78 80^ (table 2). The data type identified most by assessments was survey (consumer) (21), followed by EPS (8) and sales data (10). Data anlaysis was most often identified as quantitative (unspecified) (31) or unclear (7) (tables 3 and 4, figure 2A and B, online supplementary appendix 5).

**Table 2:** Data authors, funders and geography of assessed data

Geography	Funder of assessed data						
	British American Tobacco (subsidiary* or spokesperson†)	Imperial Tobacco/Brands (subsidiary or spokesperson)	Japan Tobacco International (subsidiary or spokesperson)	Philip Morris International (subsidiary or spokesperson)	Other TTC	More than one TTC	Funded by an organisation that has TTC members
Asia				Oxford Economics & International Tax & Investment Center^47 54 75 78 80^			
Australia	British American Tobacco (or subsidiary)^45 60^ PricewaterhouseCoopers^54 65^	Imperial Tobacco/Brands (or subsidiary)^41^		Australian Association of Convenience Stores^63^ Padilla, J, Watson, N^41 59^ Philip Morris International (or subsidiary)^41 45 56^ Roy Morgan Research^45 63^		Alliance of Australian Retailer‡ 56 58 79 Deloitte^1 45 54–60 79 80^ KPMG^41 45 54 60 68–71 78 79^	
Bulgaria				KPMG^44^	Bulgartabac^44^		
Brazil	British American Tobacco (or subsidiary)^64^						
European Union				KPMG^1 46 54 76 77 79^			
Italy				Transcrime (Università Cattolica del Sacro Cuore)^54^			
Malaysia							Confederation of Malaysian Tobacco Manufacturers^80^
New Zealand	British American Tobacco (or subsidiary)^45^ Ernst & Young^65 66^	Imperial Tobacco/Brands (or subsidiary)^45^					
Poland	ALMARES Institute for Consulting and Market Research^61^					ALMARES Institute for Consulting and Market Research^61^ KPMG^67^	Poland’s National Association for the Tobacco Industry^62^
South Africa	British American Tobacco (or subsidiary)^48^					Tobacco Institute of South Africa^48 72 73^	
UK	British American Tobacco (or subsidiary)^37 41^	Imperial Tobacco/Brands (or subsidiary)^37^	Japan Tobacco International (or subsidiary)^1 37 41^	Philip Morris International (or subsidiary)^37^ Transcrime (Università Cattolica del Sacro Cuore)^74^			Tobacco Manufacturers Association^1^
USA				Mackinac Center for Public Policy§^54^			

Grey=author.

*Subsidiary refers to a company that is controlled by a TTC but operates, usually in a different region and under a different name, eg, British American Tobacco South Africa and Souza Cruz are subsidiaries of British American Tobacco.

†Spokesperson refers to an individual who is employed by and is speaking on behalf of a TTC.

‡The Alliance of Australian Retailers is a TTC front group, funded by Philip Morris, Imperial Tobacco and British American Tobacco,^110^ so the funders of these data are listed as ‘more than one TTC’.

§The Mackinac Center for Public Policy was on Altria’s 2017 list of charitable donations and has received funding from Philip Morris dating back to the 1990s.^111 112^

TTC, transnational tobacco company.

**Table 3:** Coded data sources with descriptions & limitations

Data source	Description	Limitations of approach	Assessments identified in
Survey (tobacco consumer)	Self-reported information on illicit tobacco consumption and/or related purchase behaviours is gathered from surveying tobacco consumers. Survey data may be collected in person, by mail, online or by phone.	Questionable validity due to potential under-reporting as a result of stigma associated with illicit behaviour.^51 54^ Approach is also open to manipulation as sampling frame and survey questions may be defined with the intention to overestimate the size of the illicit market.^51^	1 44–46 48 54 55 57 58 60 65 66 68–71 74 75 78–80
Empty Pack Survey (EPS)	EPS measures non-domestic product through the collection of discarded cigarette packs and determination of their tax status.^1^	Cannot distinguish between legal and illicit non-domestic product and cannot identify counterfeits without a lab test.^54^ Additionally, it is difficult for EPS to account for tourists and commuters and estimates are narrowly limited by geographical location.^53 54^	1 37 41 46 47 54 56 60 61 68–71 75 77–80
Sales data	Many countries, usually through government agencies, hold reliable statistics about tax-paid sales of tobacco products.^51^ TTCs also hold data on sales of their own products.	Reliable tax-paid sales data in certain countries may not be publicly available^54^ and temporal biases may exist, as tax-paid sales measures tend to reflect factory or wholesale level shipments, not actual consumption.^53^	46 47 54 55 59 65 68 71 75 80
Seizure data	Data on the amount of illicit tobacco product seized by a countries’ law enforcement/customs authorities.	Can overestimate counterfeit cigarettes. As TTCs (in many jurisdictions) determine the origin of a seized product, they may identify their own products as counterfeits in order to avoid the payment of penalties.^54 113–116^ Estimates from seizure data also depend heavily on levels of enforcement—a factor which can change over time and skew results (eg, law enforcement budget increases which lead to more seizures could be misconstrued as signalling a growth in seizable product on the market).^54^	44 46 65 75 76 79 80
Expert input/opinion	Those with special insight into ITT, such as researchers and law enforcement officials, are a potential data source for information on ITT. They may be contacted for information, directly questioned, or their opinions may be presented to support a conclusion.^51^	Opinions are subjective and open to bias due to individual experience, interests, media exposure, etc.^54^	45 46 65 75 79 80
Export and import/international trade statistics	Where countries record the quantity of both imports and exports of tobacco products by country of destination, these data can be collected over time and compared with mirror image in the receiving/exporting country.^51^	Trade data do not always match correctly within a given month or year, as imports/exports may not be marked as such immediately or soon after arrival. Measures for reporting imports/exports are volatile as monetary values are subject to changes in currency exchange and volume measurements may not be consistent over time (eg, may change from weight to number of cigarettes).^54^	46 54 65 80
Survey (tobacco retailer)	Retailers may be questioned on their perceptions of issues including illicit tobacco use and availability.	Retailer surveys are not a legitimate measure of illicit tobacco trade as retailer’s perceptions of the availability of illicit tobacco are not indicative of levels of consumption and can be unreliable. Retailer surveys are also vulnerable to limitations applicable to consumer surveys.	63
Unclear	The assessment did not provide enough information to determine the data collection method/s used in the assessed data.	NA	37 44 62 64 67 72–74 76

ITT, illicit tobacco trade; NA, not applicable; TTC, Transnational tobacco company.

**Table 4:** Coded analytical methods with descriptions & limitations

Data analysis	Description	Limitations of approach	Assessments identified in
Quantitative (unspecified)	Analysis was identified as quantitative (unspecified) when assessments indicated that calculations had taken place but did not disclose the exact method used to produce them.	NA	1 37 41 45–48 54–58 60 61 63 65–73 75–80
Flows model	A method of analysis that can use multiple data sources to attempt to measure trade flows (the inflows and outflows of cigarettes) between multiple markets in order to estimate consumption.	There is currently no well-established effective flows model approach. KPMG’s ’EU Flows' model and the International Tax & Investment Center’s ’IT flows' model are examples of this approach,^19 30 31 33–35^ and have been criticised for relying on industry-provided data and methodologically weak estimates.^54^	68 71 80
Tax gap	A tax gap is the difference between the amount of tax that, in theory, should be collected and how much is actually collected. To measure this, an estimate of total tobacco consumption is produced, with legal consumption then being extracted, leaving the ‘gap’, that is, the illicit market.^117^	Cannot determine whether illicit cigarettes are counterfeit or contraband (box 1) and cannot distinguish between legal tax avoidance and illegal tax evasion.^54^	71 80 109
Econometric modelling	The use of a mathematical formula, using economic data, which considers the relationship between variables correlated with total consumption (eg, consumer income) and variables positively correlated with ITT (eg, proximity to a jurisdiction with lower price, the level of corruption, etc).^51 53^	Requires high-quality (often nationally representative data) and experienced econometricians.^54^	54 59 80
Qualitative analysis	Analysis of non-numerical information such as interviews or focus group outputs. This may involve content, narrative, discourse or framework analysis, as well as grounded theory and ethnographic approaches.	Findings cannot be generalised to larger populations and research quality is heavily dependent on the individual skills of the researcher and their agenda.^118^	45 46
Comparison of export and import statistics	Comparison of reported tobacco exports destined for a country with that country’s reported tobacco imports. Persistent discrepancies between these amounts can indicate large-scale smuggling schemes.^51^	Complicated by different countries reporting exports/imports differently (eg, in volume or monetary value) and the timing of the reporting. The trade classification system can also change over time.^54^	46
Unclear	The assessment did not provide enough information to determine the data analytical method/s used in the assessed data.	NA	37 44 59 62 64 73 74

ITT, illicit tobacco trade; NA, not applicable.

### Criticisms of industry data

Criticisms of industry data within assessments were classified in nine categories (figure 2C, table 5) and covered all aspects of the data from collection, through to analysis and presentation, as outlined in the following sections: ’Industry-funded estimates of ITT differ substantially from independent data', ’Criticisms of methodological approaches: data collection and analysis' and ’Poor presentation of results'. Only one piece of industry-funded data featured in assessments underwent a peer-review process.^81^

**Table 5:** List of coded criticisms and definitions

Criticism	Examples	Assessments identified in
Estimates were substantially higher than comparable independent estimates	Results are compared with independent estimates and found to suggest higher levels of ITT.	1 37 41 44–47 54–63 65–67 69–75 77–80
Criticism of collection methodology	Data collection method is inappropriate/unsuitable including: data collection process requires later manufacturer involvement to identify counterfeits. Measurements are defined incorrectly and/or different types of duty-not-paid products are not distinguished between during the data collection process. Data collection process does not lead to a representative sample, meaning results are not generalisable. Data collection process is not transparent.	1 37 41 44–48 54–58 60–63 65 66 68–71 75–80
Criticism of analytical method	Analytical method is inappropriate/unsuitable including: Does not account for non-response rates or sampling error. Analysis contains errors or mistakes that may influence its estimates. There is insufficient cross-validation to support findings. Analysis process is not transparent.	1 44 46–48 54 55 57 59–61 65 66 68–71 74 75 78–80
Poor presentation of results	Results are not presented adequately (eg, in a range or with CIs). Results are presented in a misleading manner. There are problems with study’s glossary/definitions. Methodological limitations are not discussed. Biased representation of existing literature.	1 37 44 46 48 54 55 57 58 60 66 67 70 71 73–76 78–80
Funding is a conflict of interest	Tobacco industry funding represents a conflict of interest.	37 44 46 48 54 62 73–77 80
Author/s do not take responsibility for findings	Authors openly distance themselves from the findings, eg, there is a disclaimer about using the results at your own risk.	54 57 60 68 71 75 78 80
Not peer-reviewed	No reference to a peer-review process.	37 54 75
Research contributes nothing of value	The research findings contribute nothing new or worthwhile to the pool of research on illicit trade.	55 74 80
Funding is not acknowledged	No acknowledgement of funding sources.	54

### Industry-funded estimates of ITT differ substantially from independent data

TTC-funded estimates on ITT were identified as being higher than comparable independent data in 31/35^1 37 41 44–47 54–63 65–67 69–75 77–80^ assessments, although one of these found that industry estimates, while higher in 11 countries than comparable independent estimates, were lower in five other countries.^77^ Only one assessment identified industry estimates as consistent with independent data.^64^ TTC-funded ITT estimates varied from 17%^80^ to 133%–337%^75^ higher than comparable independent estimates.

From assessments that identified discrepancies between industry and independent data, 27 (of 31)^1 41 44–47 54–62 65 66 69 70 72–75 77–80^ provided explanations for these. These explanations, outlined in sections ’Criticisms of methodological approaches: data collection and analysis', ’Poor presentation of results' and ’Transparency and replicability', were also mentioned in assessments that did not compare industry and independent estimates.

### Criticisms of methodological approaches: data collection and analysis

The majority of assessments (29/35) criticised industry-funded data collection.^1 37 41 44–48 54–58 60–63 65 66 68–71 75–80^ The issues identified primarily focused on the data collection method’s (un)suitability for measuring illicit and failure to provide representative samples (table 5). In particular, assessments criticised the use of EPS, which cannot reliably distinguish between illegal (illicit) and legal forms of non-domestic product (table 3), to measure illicit^41 45 46 54 60 80^ and consumer surveys, due to both potential under-reporting (table 3) and over-reporting of particular types of illicit trade.

#### Empty Pack Surveys

EPS were also criticised for focusing on urban areas, where illicit consumption is likely to be higher,^54 69 75 78 80^ and over-representing litter in public places^41 68^ and thus packs smoked by those most likely to litter, such as tourists, students^41 60^ and users of illicit tobacco product.^41^ Other assessments stated that information required to determine the representativeness of EPS was not provided^37 47 61 65 77^ and unexplained changes were made during the sampling process.^61^

#### Consumer and retailer surveys

Industry-commissioned consumer surveys were criticised for their sampling approach,^26 27 48 54 66 69 78 80 82^ including relying on non-random samples where participants, already on a market research email database, opt in to conduct an online survey^69–71 78 80^ and for having low response rates with no attempt to correct for this or to establish the representativeness of the sample.^54 55^

Assessments identified overlap around terms used in surveys, which may have led to responses being double-counted, for example, counterfeit and contraband.^55 56 80^ As counterfeit products are a form of contraband (box 1), a survey that asks separately about counterfeit and contraband is likely to lead to the counterfeit product being reported more than once (as both counterfeit and contraband), incorrectly inflating levels of illicit.^80^ Similarly, because it can be difficult for consumers to differentiate between legal and illicit products (eg, survey respondents may not know the tax-paid status of tobacco products they have purchased and have assumed that cheap cigarettes they had tried were counterfeit or contraband despite this not being the case), consumer surveys may lead to legal consumption being falsely reported as illicit.^45 55 57 79^

A retailer survey was criticised for how it presented a question regarding illicit tobacco, simply asking if participants had ‘seen, read or heard about’ illicit tobacco products. Given frequent media interest in illicit tobacco, it is likely that respondents would answer yes to this question. Even if this question had been presented differently, surveys of retailer’s perspectives on illicit tobacco availability are not indicators of illicit tobacco trade as perceived availability of illicit products is not evidence of illicit consumption.^63^

#### Problems with data analysis

Problems with the analytical process were identified in 22/35^1 44 46–48 54 55 57 59–61 65 66 68–71 74 75 78–80^ of assessments. These included errors in how averages were calculated which would overestimate ITT,^68 70 71^ not including sensitivity analyses in the modelling to illustrate how estimates might differ if assumptions made in a modelling process changed,^65 66^ and relying solely on industry data to produce model-estimates^44 47 61 79^ and cross-validate findings^46 54^ when other data were available. In one case, it was argued that an industry-funded study, based on econometric modelling (see table 4 for definition), applied assumptions that were unlikely to be accurate.^59^ In another, a BAT-sponsored website extrapolated illicit estimates from a five-city survey to the whole of Australia, failing to account for likely differences in availability of illicit tobacco between remote Australian towns and major cities thus undermining accuracy.^60^ 

 One assessment identified a methodological change between KPMG’s 2011 and 2012 Project Star reports, where a pack-based measure was replaced with a cigarette-based measure, leading to an artificially higher estimate compared with previous years.^1^ In particular, this methodological change was difficult to identify and appears to have been applied just in some countries where novel tobacco control policies were being discussed (eg, standardised packaging in the UK).^1^

### Poor presentation of results

#### Missing information

Issues with how findings were presented were identified in 21 (60%) assessments.^1 37 44 46 48 54 55 57 58 60 66 67 70 71 73–76 78–80^ Some identified TTC-funded reports that lacked confidence intervals or margins of error required to interpret the accuracy and significance of estimates.^54 70 71 75 78^ Others reported that TTC-funded reports failed to highlight potentially embarrassing findings for TTCs. These include tobacco industry illicit (box 1) comprising the majority of the illicit market in studied regions^80^ and the identification of substantial reductions in consumption that contradict industry narratives that increased taxation increases ITT.^79^

#### Misrepresentation of findings

Assessments identified several examples of data seemingly being deliberately misrepresented in TTC-funded reports or by TTCs directly, whereby data on illicit tobacco were presented as a proportion of total tobacco consumption. This gives the false impression that the illicit market has increased when in absolute terms, both are falling with consumption declining at a faster rate.^54 67^ This is increasingly problematic, with global consumption expected to continue declining.^83^ Additional examples include downwards-adjustment of previous estimates to create the impression that illicit trade is growing^37 48^ and TTCs potentially overclassifying illicit cigarettes as counterfeit.^76^

#### Misrepresentation of pre-existing data

Assessments suggested that independent data were misrepresented in tobacco industry reports, with selective presentation of available estimates; with lower estimates not featuring in industry data and reports^46 74 84^; presented estimates being inconsistent over time^73^; government estimates being represented through the highest estimates offered rather than the most likely^1^ and claims citing independent data being indeterminable from the cited data.^58^

### Transparency and replicability

Bringing together the criticisms overall, industry-funded data were criticised for a fundamental lack of transparency at every stage of the research process, from sampling and data collection through analysis to publication of findings. Descriptions of EPS lacked information on sample frames, where and when data were collected, how legal and illegal packs were distinguished or any methodological details at all.^1 37 46–48 56 69 75 77 78^

Consumer surveys were criticised for not providing response rates,^65 68 71 75^ important details of the sample population such as smoking characteristics^70^ and on the wording and sequencing of the questions asked.^57^ Industry-funded analytical methods were criticised for lacking transparency,^46 54 66 75^ with the IT flows model, used by International Tax & Investment Center and Oxford Economics,^33–35^ relying on other models created by Oxford Economics that are not clearly outlined.^75 80^

As demonstrated in the ’Poor presentation of results' section, assessments also identified a lack of transparency with how their findings and the findings of others were presented. For example, some industry-funded reports highlighted increasing illicit consumption in certain countries within a region while omitting contrary examples from within the same region being researched (table 6).^75 80^

**Table 6:** Qualitative examples of criticisms related to transparency

Associated critique	Example taken from an assessment
Data collection	"Despite internet searches and multiple attempts to contact the tobacco manufacturers and the research company, we do not have all details of the method used by the tobacco industry. For example, we lack information on how the sample paths and bins for the discarded pack collection were selected, what pack features were taken into account when deciding whether the pack is tax-paid or non-tax-paid. We only know that the packs were examined by the four respective producers to find counterfeit cigarettes".^61^
Data analysis	’There is limited information to explain how the model captures the various factors that influence consumption and insufficient information to independently replicate the report’s estimates. In addition, the model is applied inconsistently in each country'.^80^
Presentation of results	’Different sources and methods are used across countries, leading to results that are not comparable to one another, yet presented for comparison, without acknowledgement of their distinctions'.^75^

## Discussion

Findings from this review demonstrate that concerns raised with industry-commissioned reports produced by such organisations are widespread.^1 41 44–47 54 60 61 68–71 75–80^ Our findings suggest that TTC-funded data routinely overestimate illicit, feature substantial methodological problems and fail to meet the standards of accuracy and transparency that are set by high-quality research publications. The consistency with which these issues have been identified in TTC-funded reports, and a failure for industry-funded reports to make their research more transparent for the purpose of replicability, may indicate that the tobacco industry is deliberately producing misleading data on ITT. Even in cases where suitable independent data were publicly available,^54^ industry-sourced data such as sales and prevalence figures were used both to produce estimates in industry-commissioned reports and to attempt to cross-validate them.^44 46 75^

The main strength of this research is that it is the first attempt to systematically identify and review literature that assesses the quality of industry data on ITT. It has made extensive efforts to identify academic research and grey literature, critically appraised this literature before double-coding it in depth, presented findings and relevant contextual information in an accessible manner and provided an overview of ongoing concerns with TTC-funded data on ITT.

However, as only assessments written in English were featured, it is possible that relevant literature in other languages was excluded. Furthermore, the findings of this work are determined by the underlying literature used and may be limited by its accuracy, quality and any potential publication bias. In relation to this last point, while we included all independent assessments (positive or negative), of TTC-funded data it is possible that such assessments focus almost exclusively on data/reports that are problematic.

It is widely recognised that no currently available method for assessing ITT is flawless. It should also be noted that the appropriateness of a method and the usefulness of data resulting from it is dependent on the research question/s being considered by a study. However, the methodologies identified by the assessments can and have been used effectively by multiple non-industry sources. For example, EPS have been the focus of several well-executed measurements of tax avoidance and evasion by independent researchers^8–10^; much of what is known about adult users of illicit tobacco is based on self-reported information collected through both large population and localised surveys^3^; econometric modelling has been used extensively to measure ITT, primarily in the USA, for decades^3^ and a tax gap approach is currently used by the UK government to estimate levels of ITT.^11–15^

Concerns regarding the representativeness and objectivity of data collection methodologies, errors and mistakes in the data analysis, and poor presentation of results, suggest that the quality of industry data on ITT as a whole is below the expected standard to be considered reliable. Together, all of these problems may help explain the disparity between industry-funded and independent estimates of ITT.

Taken together, this indicates that it is how methods are employed and who employs them that dictates the quality of their output. Improving the reliability of estimates on ITT does not therefore mean rejecting available methodologies but ensuring they are used appropriately and transparently. Our findings suggest that industry-funded research has routinely failed to meet these standards.

Our findings correspond with the tobacco industry’s long history of manipulating research, including its extensive efforts to undermine and cause confusion on science showing the negative health impacts of smoking^85 86^ and second-hand smoke,^87^ and suggest that similar strategies are now being used by TTCs in relation to ITT. Despite overwhelming evidence of the TTCs’ historical complicity in tobacco smuggling,^43 88–94^ the tobacco industry now portrays itself as key to solving the ITT^91^ and presents its funding of research on ITT as its attempt to reduce the societal burden of illicit trade and organised crime.^49^ However, this review’s findings demonstrate that the contribution of tobacco industry-funded data on ITT thus far in aiding understanding of ITT is extremely limited, if not counterproductive.

The primary purpose of tobacco industry-funded data on ITT seems to be to serve as a platform for the industry’s lobbying and public relations strategies. With the recent growth in TTC-funded reports on ITT,^95–97^ their widespread coverage in the media^98–100^ and the establishment of PMI IMPACT^50^—putting US$100 million for research on ITT—this situation will only worsen. A similar campaign may now be under way in the field of harm reduction with PMI pledging US$80 million annually for the next 12 years to fund the Foundation for a Smoke-Free World, which claims to ‘advance smoking cessation and harm-reduction science and technology’.^101 102^

Our findings suggest that a more effective approach to obtaining accurate research on illicit tobacco would be to tax tobacco companies and independently administer the funding thus raised based on previously developed models^103^ that have been successfully used in Thailand and California.^104^

In the meanwhile, existing independent assessments make it clear that TTC-funded data on ITT cannot be trusted. By identifying all of the most common criticisms levelled against industry-funded data on ITT, our findings compliment Ross’ criteria for assessing the quality of estimates on tobacco tax avoidance and evasion^54^ on ITT and can therefore be used as a framework to assess the quality of future TTC-funded studies on the ITT. It is hoped that this will aid others in determining the quality of future TTC estimates in a sufficiently timely manner to contribute to policy debates.

**What this paper adds**

- Transnational tobacco companies (TTCs) produce and publicise data on the illicit tobacco trade (ITT), which is then used to influence policymakers.^37–44^
- This is the first paper to systematically review assessments of TTC-funded data on the ITT.
- It finds that TTC-funded data covering multiple world regions routinely exaggerate/overestimate levels of illicit when compared with independent sources and that this is a result of problems at all stages in the research process, with inappropriate usage of methods of data collection and data analysis, misleading presentation of results and a lack of transparency throughout, with information necessary for replication often being excluded.
- The review concludes that TTC-funded data on ITT cannot be trusted and argues that if the global scale of the ITT is to be better understood, more high-quality and transparent ITT research is needed, and a potential means for providing this would be a tax on tobacco companies, with a portion of raised funds going towards independent development of established methodologies.
