# Supplementary material for: Tobacco industry data on illicit tobacco trade: a systematic review of existing assessments
Source: Tob Control. 2018 Aug 22;28(3):334–45. doi: 10.1136/tobaccocontrol-2018-054295 (PMC6580768; doi:10.1136/tobaccocontrol-2018-054295)
Supplement: Supplementary file 1 [file tobaccocontrol-2018-054295supp001.pdf]

Appendix 1: Review protocol
Review question/objective:

The objective of this systematic review is to assess the quality of data presented in Trans-national Tobacco Company (TTC)-funded data on the illicit tobacco trade (ITT). This will be achieved by identifying and reviewing pre-existing assessments tobacco industry data on ITT. These will then be summarised to provide a substantive overview of the quality of TTC data on the ITT and to determine the nature of any problems identified, thereby filling a gap in the existing knowledge base.

The specific review question to be addressed:

What does the existing literature tell us about the quality of TI data on ITT?

Further questions that the review question should engage with:

What problems, if any, have been identified with TI data on the ITT and how can these best be categorised?

How does TI data on illicit trade compare with independent data?

Inclusion/Exclusion Criteria

Inclusion Criteria:

-Academic and grey literature in printed or electronic formats.
- Clearly identifies the specific study/ies being assessed.
-The study/ies being assessed must be linked to a TTC- If it's not clear in the study that the assessed study is industry linked, the researcher will identify this.
- The work provides an original assessment i.e. does not solely refer to pre-existing assessments.
-Assessment is written in English.
-Assessment did not receive funding from the tobacco industry.
-Is not a duplicate

Comprehensive data search

Database Sources

Web of Science, Embase, Pubmed & Pubmed Central, International Bibliography of Social Sciences (IBSS), ScienceDirect, and Business Source Complete will all be searched as well as specialty journals (Addiction, Health Economics, and Tobacco Control).

Search Strands

In order to find papers to include within this systematic review, searches will be undertaken using the following strands, based on the advice of a qualified librarian.

Main strand:

(("Philip Morris" OR "PMI" OR "British American Tobacco" OR "BAT" OR "Imperial Tobacco" OR "Imperial Brands" OR "Imperial" OR "ITG" OR "Japan Tobacco" OR "JTI" OR "Tobacco company" OR "transnational tobacco company" OR "TTC" OR "TTCs") AND ("research" OR "evidence" OR data* OR "study" OR "studies" OR report*) AND (illicit* OR illegal* OR smuggl* OR "contraband" OR counterfeit*) AND ("tobacco" OR cigar*)).

Multiple variations of this strand will be used in order to identify the most effective for each database.

Non-database Sources

Google searches will be conducted in order to identify grey literature. Searches on 'illicit tobacco' and the names of prominent TTCs (British American Tobacco, Imperial Tobacco/Brands, Philip Morris International, Japan Tobacco International) will be used as well as an 'allintitle' search of 'illicit tobacco. Searches of 'illicit tobacco' will also be performed on websites of organisations engaged in industry monitoring on this topic. In conjunction with other researchers, a list of appropriate experts was created. Each will be asked if they know of any further relevant literature to include and experts to contact. The list includes persons who have previously conducted research on the ITT, tobacco control experts, members of NGOs, and non-academics who have written on the topic. The bibliographies from collected papers that meet the inclusion criteria will be hand-searched to identify any additional literature.

Synthesis of data

Each search will be documented in a database. Recorded details include the date of search, source/database used, subsection i.e. search type, the year from and to of the search, the search terms use and the amount of results found. All of the selected literature was stored in a specific library within a reference management system (Endnote). A record of the total number of included studies at each stage of the systematic review will be completed throughout the process, and the results will be summarised in a chart in the final report.

A data extraction and critical appraisal form has been developed in accordance with all authors in order to capture important characteristics of the collected papers and the industry data they address. This includes the author/s, year of publication, type of publication (eg. academic journal, NGO report), and brief outlines of the aims of each paper as well as the title, year of publication, and producers of the industry data that is assessed. The critical appraisal process for the assessments themselves considered whether they underwent a peer-review process, disclosed their funding sources, and outlined their methodological approach. In order to avoid bias, the inclusion/exclusion criteria and data extraction form will be piloted with another researcher before all of the data is collected and analysed. After data collection the coding framework was be piloted using 3 papers identified during by the search process. Any discrepancies will be documented and agreement will be reached between the researchers. Any changes made to the framework as a result of these discussions will also be noted.

100% of the included assessments will be second-coded independently by an additional author (either 2nd or 3rd author) and inter-coder reliability will be calculated. All disagreements will be documented in an Excel file and fully-resolved between the coders. Throughout the coding process, NVivo 10 will be used to collect qualitative statements from assessments that are applicable to categories within the coding framework.

Appendix 2. List of identified assessments				
**Title**	**Author/s**	**Year**	**Source**	**Reference**
A mountain or a molehill: is the illicit trade in cigarettes undermining tobacco control policy in South Africa?	Blecher, E.	2010	JOURNAL: Trends in Organized Crime	77
Plain Packaging of tobacco products: a review of the evidence	Cancer Council Victoria	2011	REPORT: Cancer Council Victoria	63
Comments on survey of Australian retailers' views about plain packaging	Cancer Council Victoria	N/A	REPORT: Cancer Council Victoria	68
Questions and answers on plainpackaging in Australia, Facts sheet no. 3	Cancer Council Victoria	2016	FACT SHEET: Cancer Council Victoria	83
Removing the emperor's clothes: Australia and tobacco plain packaging	Chapman, S., Freeman, B.	2014	EBOOK	65
Did the tobacco industry inflate estimates of illicit cigarette consumption in Asia? An empirical analysis	Chen, J., McGhee, S.M., Townsend, J., Lam, T. H., Hedley, A. J.	2015	JOURNAL: Tobacco Control	47
The Market for Legal and Illegal Cigarettes in Poland: A Closer Look at Demand and Supply Side Characteristics	Ciecierski, C.	2007	WORKING PAPER	67
Will Plain Packaging Reduce Cigarette Consumption?	Clarke, H., Prentice, D.	2012	JOURNAL: Economic Papers	64

‘It will harm business and increase illicit trade': an evaluation of the relevance, quality and transparency of evidence submitted by transnational tobacco companies to the UK consultation on standardised packaging 2012	Evans-Reeves, K., Hatchard, J., Gilmore, A.B.	2015	JOURNAL: Tobacco Control	37
Illicit trade, tobacco industry-funded studies and policy influence in the EU and UK	Fooks, G.J., Peeters, S.,Evans-Reeves, K.	2013	JOURNAL: Tobacco Control	79
Towards a greater understanding of the illicit tobacco trade in Europe: a review of the PMI funded 'Project Star' report	Gilmore, A.B., Rowell, A., Gallus, S., Lugo, A., Joossens, L., Sims, M.	2013	JOURNAL: Tobacco Control	46
Estimating the size of illicit tobacco consumption in Brazil: findings from the global adult tobacco survey	Iglesias, R.M., Szklo, A.S., de Souza, M.C., de Almeida, L.M.	2016	JOURNAL: Tobacco Control	69
Assessment of the European Union's illicit trade agreements with the four major Transnational Tobacco Companies	Joossens, L., Gilmore, A.B., Stoklosa, M., Ross, H.	2015	JOURNAL: Tobacco Control	81
Illicit cigarettes and hand-rolled tobacco in 18 European countries: a cross-sectional survey	Joossens, L., Lugo, A., La Vecchia, C., Gilmore, A.B, Clancy, L., Gallus, S.	2013	JOURNAL: Tobacco Control	82
Illicit Tobacco Trade: Monitoring and Mitigating Risk in New Zealand	Paynter, J., Joossens, L.	2010	REPORT: Action on Smoking and Health (ASH)	70
Illicit trade of tobacco in Australia. A report prepared by Deloitte for British American Tobacco Australia Limited, Philip Morris Limited and Imperial Tobacco Australia Limited: A critique prepared by Quit Victoria, Cancer Council Victoria, March 2011	Quit Victoria	2011	REPORT: Cancer Council Victoria	62

Illicit trade of tobacco in Australia. A report prepared by Deloitte for British American Tobacco Australia Limited, Philip Morris Limited and Imperial Tobacco Australia Limited: A critique prepared by Quit Victoria, May 2012	Quit Victoria	2012	REPORT: Cancer Council Victoria	55
Analysis of KPMG LLP report on use of illicit tobacco in Australia: 2013 Half Year Report	Quit Victoria	2014	REPORT: Cancer Council Victoria	73
How big a problem is illicit tobacco and has it increased since the introduction of plain packaging in Australia- a critique of the KPMG October 2014 half-year report on illicit tobacco in Australia and compilation of data from ACBPS seizures and data from survey of smokers and retail audits	Quit Victoria	2015	REPORT: Cancer Council Victoria	74
Analysis of KPMG LLP report on use of illicit tobacco in Australia: 2014 Full Year Report, 30th March 2015	Quit Victoria	2015	REPORT: Cancer Council Victoria	75
Analysis of KPMG LLP report on use of illicit tobacco in Australia 2013 Full year report	Quit Victoria	2014	REPORT: Cancer Council Victoria	76
Understanding and measuring cigarette tax avoidance and evasion: a methodological guide	Ross, H.	2015	REPORT: Tobacconomics	59
Tobacco industry manipulation of data on and press coverage of the illicit tobacco trade in the UK	Rowell, A., Evans-Reeves, K., Gilmore, A.B.	2014	JOURNAL: Tobacco Control	1

Availability of illicit tobacco in small retail outlets before and after the implementation of Australian plain packaging legislation	Scollo, M., Bayly, M., Wakefield, M.	2014	JOURNAL: Tobacco Control	60
Tobacco in Australia, facts and issues: 13.7.8.4, Avoidance and evasion of taxes on tobacco products	Scollo, M., Winstanley, M.H.	2016	EBOOK: Cancer Council Victoria	84
Use of illicit tobacco following introduction of standardised packaging of tobacco products in Australia: results from a national cross sectional survey	Scollo, M., Zacher, M., Coomber, K., Wakefield, M.	2015	JOURNAL: Tobacco Control	61
Early evidence about the predicted unintended consequences of standardised packaging of tobacco products in Australia: a cross-sectional study of the place of purchase, regular brands and use of illicit tobacco	Scollo, M., Zacher, M., Durkin, S., Wakefield,M.	2014	JOURNAL: Tobacco Control	41
Change in tobacco excise policy in Bulgaria: the role of tobacco industry lobbying and smuggling	Skafida, V., Silver, K.E., Rechel, B.P.D., Gilmore, A.B.	2014	JOURNAL: Tobacco Control	44
Asia-11 Illicit Tobacco Indicator 2012: More Myth than Fact, A Critique by SEATCA	Southeast Asia Tobacco Control Alliance (SEATCA)	2014	REPORT: Southeast Asia Tobacco Control Alliance (SEATCA)	85
Failed: A Critique of the ITIC/OE Asia-14 Illicit Tobacco Indicator 2013	South East Asia Tobacco Control Alliance (SEATCA)	2015	REPORT	80
Is the illicit cigarette market really growing? The tobacco industry's misleading math trick	Stoklosa, M.	2015	JOURNAL: Tobacco Control	72

Contrasting academic and tobacco industry estimates of illicit cigarette trade: evidence from Warsaw, Poland	Stoklosa, M., Ross,H.	2013	JOURNAL: Tobacco Control	66
Review of Ernst & Young's Report on New Zealand's Illicit Tobacco Market	Tailor, N., Branson, J.	2010	REPORT: NZIER (for ASH)	71
Measuring changes in the illicit cigarette market using government revenue data: the example of South Africa	Van Walbeek, C.	2014	JOURNAL: Tobacco Control	78
Are the tobacco industry's claims about the size of the illicit cigarette market credible? The case of South Africa	Van Walbeek, C., Shai, L.	2014	JOURNAL: Tobacco Control	48

Appendix 3: inclusion, data extraction, and critical appraisal form

Review of assessments of tobacco industry data on the illicit tobacco trade: inclusion, data extraction, and critical appraisal form

Full reference		
First reviewer		
Second reviewer		
Check list for inclusion		
(Title and abstract check) Is the study (a) in English?	Yes	No
(Title and abstract check) Does a) feature discussion of and/or include data on illicit trade?	Yes	No (exclude from review)
Does a) assess tobacco industry data on illicit trade (b)?	Yes	No (exclude from review)
Does a) clearly identify the source/s of this data?	Yes	No (exclude from review)
Does a) provide an original assessment of tobacco industry data on illicit trade?	Yes	No (exclude from review)
Did a) receive tobacco industry funding?	Yes (exclude from review)	No
Data Extraction		
What are the aims of a), as interpreted by the reviewer?		
What are the aims of a), according to the words of a)?		
What identifying information does a) provide regarding b)? Organisation/title/year/on behalf of/industry data source (if different from producer/s or report)		
According to a), which regions/countries/localities does b) focus on? Region/Country/Locality-City		

According to a), what are the key claims about illicit trade that b) makes?		
Critical appraisal of a)		
Did a) undergo a peer-review process	Yes	No
Does a) disclose its funding source/s?	Yes	No
Does a) outline its methodological approach?	Yes	No
Notes		

Appendix 4: Coding framework

**2.6 According to a), which of the following most-accurately represents b)'s data types?**	**Options: Yes - a) identifies this as a data type used by b)<br>No - a) does not identify this as a data type used by b)**
**a Export and import/international trade statistics**	**Country-reported data on exports of tobacco by country of destination and of imports of tobacco by country of origin**
**b Empty Pack Survey (EPS)**	**Data taken from the collection of discarded cigarette packs which are assessed and categorised**
**c Survey (illicit consumption)**	**Includes survey data collected in person, by mail, online and by phone. This includes an assessment of smokers' packs in person-to-person survey which involves interviewers asking passing smokers to show their cigarette packs which are then assessed and categorised, consumers sending in empty packs in exchange for free gifts which are then assessed and categorised. It also includes survey data of tobacco users' self-reported illicit tobacco consumption and/or purchase behaviours**
**d Smoker and non-smoker survey on knowledge of illicit tobacco**	**Data from survey about participant's own views on the prevalence of illicit tobacco in their area**
**e Undercover Test Purchases (UTP)**	**Data collected by individuals who attempt to purchase illicit tobacco while not revealing their aim**
**f Sales figures**	**Sales data provided by tobacco companies**
**g Seizure figures**	**Seizure data provided by law enforcement ad customs organisations**
**h Expert input**	**Data collected by direct questioning of, or stating the opinions of: smuggling researchers/experts on illicit tobacco; individuals who have illicit tobacco experience, either current of previous, within law enforcement; tobacco retailers; OR from published reports containing estimates of smuggling based on a canvass of experts who are familiar with local market conditions e.g. World Tobacco File**
**i Survey (retailers)**	**When retailers are questioned on their perceptions of issues including illicit tobacco use and availability. Survey data may be collected in person, by mail, online or by phone.**
**j Unclear**	**it is unclear from a) what data types b) used**
**2.7 According to a), what analysis methodologies did b) use?**	**Options: Yes - a) identifies this as an analysis methodology that b) used<br>No - a) does not identify this as an analysis methodology that b) used**

**a Tax gap method**	**Estimate of total consumption - legitimate consumption = illicit market.**<br><br>**e.g. "consumption is estimated, from which legitimate consumption is subtracted, the remainder being the illicit market"**
**b Econometric modelling**	**An econometric study (a mathematical formula using economic data): An econometric model specifies the statistical relationship that is believed to hold between the various economic quantities pertaining to a particular economic phenomenon under study. In the case of illicit trade, of the relationship between observed tax paid sales, variables associated with the demand for tobacco, and variables associated with smuggling.**
**c Quantitative analysis**	**Analysis that is based on unique (differs from either the tax gap method or econometric modelling methods) mathematical and statistical modelling.**<br><br>**e.g. 'The mathematically correct way to calculate the average amount of unbranded tobacco used each year is to estimate the quantity that each (unbranded tobacco) smoker smoked per year (by multiplying their quantity with their frequency of purchase) and to then divide by the number of smokers purchasing unbranded tobacco.'.**<br><br>**total consumption = quantity x frequency of purchase**<br><br>**÷ number of consumers**<br><br>**= average amount of unbranded tobacco used each year**<br>**Any other form of quantitative analysis, such as descriptive statistics of survey data collected over time.**
**d Qualitative analysis**	**Analysis of non-numerical information such as interview or focus group output. This may involve content, narrative, discourse, or framework analysis, as well as grounded theory and ethnographic approaches.**
**e Assumptions about interception rates based on customs data**	**Examination of the annual detainment of cigarettes and loose tobacco by Customs using data from Customs (airport, shipping, mail).**<br><br>**e.g. 'Ernst & Young use the average amount of tobacco (2007 to 2009) detained by New Zealand Customs and make assumptions about interception rates to estimate the amount which may have entered New Zealand without excise duty being paid'**
**f Comparison of export and import statistics**	**Comparison of reported tobacco exports destined for a country with that country's reported tobacco imports. Persistent discrepancies between these amounts provide an estimate of the amount of wholesale smuggled tobacco**

**g Flows Model**	**A method of analysis that measures trade flows (the inflows and outflows of cigarettes) between multiple markets in order to estimate consumption. KPMG's EU flows model and the International Tax & Investment Centres' (ITIC) IT Flows Model are examples of this approach.**<br><br>**Outflows of duty-paid cigarettes to other markets (Legal domestic consumption)**<br>**+ non-domestic legal to legal domestic sales to derive total legal consumption**<br>**then estimate total illicit consumption to derive total consumption, using EPS for non-domestic illicit and retail audits for domestic illicit.**<br><br>**"The IT Flows model uses an estimate of legal Non--Domestic consumption that is generated with the help of the OE Tourism Model. This model is only vaguely described and readers have to speculate as to how the model's "over 50,000 indicators" were used to estimate the number of tourists, and why it is relevant that the model forecasts 10 years into the future if the estimates in the report are for 2013"**
**h Unclear**	**b)'s analysis methodology is not specified or sufficient details need to determine b)'s analysis methodology have not been provided.**
**2.8 What criticisms does a) make about b)?**	**Options: Yes - a) provides a critique on this area.**<br>**No - a) does not provide a critique on this area**<br>**No (Praise) - a) does not provide a critique, instead praising b) on this area.**
**a Not peer-reviewed**	**a) states that there is no reference to a peer-review process in b) and/or specific terms under which b) was prepared are not disclosed.**<br><br>**e.g. 'there is no reference to a peer-review process**
**b Funding is not acknowledged**	**a) states that b) does not acknowledge or disclose its funding sources**
**c Author/s do not take responsibility for findings**	**a) claims that b)'s authors do not claim responsibility for and/or try to separate themselves from the research and its findings and/or there is a disclaimer about using the results at your own risk.**

**d Criticism of data collection methodology**	**a) identifies problems with method/s of data collection used in b). This may include:**<br><br>**•the data collection process failing to account for factors that could influence the scope of illicit trade in b)'s final estimates**<br><br>**•b) featuring a data collection method that is not appropriate for what b) aims to measure**<br><br>**•issues with the representativeness and generalisability of b)'s data collection process**<br><br>**•problems with the transparency and replicability of b)'s data collection process**<br><br>**• manufacturer involvement in the data collection process (through counterfeit identification)**<br><br>**•measurements being defined incorrectly and/or different types of illicit not being distinguished between during the data collection process by b)**<br><br>**•b)'s data collection process preferring subjective over objective criteria (e.g. self-reporting over objective criteria)**
**e Criticisms of analysis methodology**	**a) identifies problems with the analysis methodology/ies used in b). This may include:**<br><br>**• the process failing to account for factors that could influence the scope of illicit trade in b)'s final estimates**<br><br>**• b) using a method of analysis that is not suitable for b) is attempting to analyse, issues with the representativeness and generalisability of b)'s analysis methodology**<br><br>**• problems with the transparency and replicability of b)'s analysis methodology**<br><br>**• b)'s analysis methodology contains errors or mistakes that may influence its estimates**<br><br>**• insufficient cross-validation by b) to support its findings**

**f Poor presentation of results**	**a) identifies problems with how b) presents its results. This may include:<br><br>• b)'s results not being presented adequately (in a range or with confidence intervals)<br><br>• b)'s results are presented in a misleading manner (the size of the illicit market is expressed as a share of the licit market, which makes it look bigger)<br><br>• b) does not acknowledge its methodological weaknesses<br><br>•or b) over-looks pre-existing research that undermines or contrasts with its main arguments and instead relies on pre-existing data that supports its positions<br><br>• b) provides unsubstantiated critiques of pre-existing research that undermine b)'s findings and/or design.<br><br>•There are issues with terminology and definitions used in b)'s glossary**
**g Findings are not significant/have no impact**	**a) claims that b)'s findings contribute nothing new or worthwhile to the pool of research on illicit trade.**
**h No clear research goals**	**a) claims that b) does not clearly state its research aims/goals**
**I Funding is a conflict of interest**	**a) claims that b)'s funding source may have influenced the quality of b)'s approach and/or b)'s data**
**j Estimates differ substantially from independent estimates**	**a) compares b)'s results with independent estimates and claims that there are significant differences. Where comparisons are used to develop criticisms of a specific feature of b), they should be coded in the relevant category (criticism of methodology, poor presentation of results, etc.).**
**ROUND THREE- Critical appraisal of a)**	
**3.1 Did a) undergo a peer-review process**	
**Yes**	**a) featured in a peer-reviewed journal**
**Unclear**	**a) did not feature in a peer-reviewed journal but refers to a peer-review process having been undertaken**
**No**	**a) did not feature in a peer-reviewed journal and does not refer to a peer-review process having taken place**
**3.2 Does a) disclose its funding source?**	
**Yes**	**a) discloses if it was commissioned by an external party and, if so, identifies this party and its funding sources.**
**No**	**a) does not disclose if it was commissioned by an external party or discloses external funding but does not identify the party**

**3.3 Does a) outline it's methodological approach?**	
**Yes**	**a) features a dedicated outline of its method of analysis**
**No**	**a) does not feature a dedicated outline of its method of analysis**

Appendix 5: Coded methodologies and criticisms

		Criticism									
	**Method**	**Inflated estimates (31)**[1, 37, 41, 44, 46, 47, 55, 59-68, 70-72, 74-80, 82-85]	**Flawed collection methodology (29)**[1, 37, 41, 44, 46-48, 55, 59-63, 65-68, 70, 71, 73-76, 80-85]	**Flawed analysis methodology (22)**[1, 44, 46-48, 55, 59, 62, 64-66, 70, 71, 73-76, 79, 80, 83-85]	**Poor presentation of results (21)**[1, 37, 44, 46, 48, 55, 59, 62, 63, 65, 71, 72, 75, 76, 78-81, 83-85]	**Lacks Transparency (19)**[1, 37, 46-48, 59, 60, 62, 66, 70, 71, 73-76, 80, 82, 83, 85]	**Funding a conflict of interest (12)**[37, 44, 46, 48, 59, 67, 78-82, 85]	**Author/s do not take responsibility for findings (8)**[59, 62, 65, 73, 76, 80, 83, 85]	**Not peer-reviewed (3)**[37, 59, 80]	**Research adds nothing of value (3)**[55, 79, 85]	**Funding not acknowledged (1)**[59]
**Data Collection**	**Survey (illicit consumption) (21)**[1, 44, 46, 48, 55, 59, 61-63, 65, 70, 71, 73-76, 79, 80, 83-85]	19[1, 44, 46, 55, 59, 61-63, 65, 70, 71, 74-76, 79, 80, 83-85]	20[1, 44, 46, 48, 55, 59, 61-63, 65, 70, 71, 73-76, 80, 83-85]	19[1, 44, 46, 48, 55, 59, 62, 65, 70, 71, 73-76, 79, 80, 83-85]	17[1, 44, 46, 48, 55, 59, 62, 63, 65, 71, 75, 76, 79, 80, 83-85]	14[37, 46, 48, 59, 62, 70, 71, 73-76, 80, 83, 85]	8[37, 44, 46, 48, 59, 79, 80, 85]	8[59, 62, 65, 73, 76, 80, 83, 85]	3[37, 59, 80]	3[55, 79, 85]	1[59]
	**Empty Pack Surveys (EPS)(18)**[1, 37, 41, 46, 47, 59, 60, 65, 66, 73-76, 80, 82-85]	18[1, 37, 41, 46, 47, 59, 60, 65, 66, 73-76, 80, 82-85]	18[1, 37, 41, 46, 59-61, 65, 66, 73-76, 80, 82-85]	14[1, 46, 47, 59, 65, 66, 73-76, 80, 83-85]	11[1, 37, 46, 59, 65, 75, 76, 80, 83-85]	15[1, 37, 46, 47, 59, 60, 66, 73-76, 80, 82, 83, 85]	6[37, 46, 59, 80, 82, 85]	6[59, 65, 73, 76, 83, 85]	3[37, 59, 80]	1[85]	1[59]
	**Sales Figures (10)**[46, 47, 55, 59, 64, 70, 73, 76, 80, 85]	9[46, 47, 55, 59, 64, 70, 76, 80, 85]	9[46, 47, 55, 59, 70, 73, 76, 80, 85]	10[46, 47, 55, 59, 64, 70, 73, 76, 80, 85]	6[46, 55, 59, 76, 80, 85]	8[46, 47, 59, 70, 73, 76, 80, 85]	4[46, 59, 80, 85]	5[59, 73, 76, 80, 85]	2[59, 80]	2[55, 85]	1[59]
	**Seizure Figures (7)**[44, 46, 70, 80, 81, 84, 85]	6[44, 46, 70, 80, 84, 85]	7[44, 46, 70, 80, 81, 84, 85]	6[44, 46, 70, 80, 84, 85]	6[44, 46, 80, 81, 84, 85]	4[46, 70, 80, 85]	5[44, 46, 80, 81, 85]	2[80, 85]	1[80]	1[85]	0
	**Expert Input (6)**[46, 61, 70, 80, 84, 85]	6[46, 61, 70, 80, 84, 85]	6[46, 61, 70, 80, 84, 85]	6[46, 61, 70, 80, 84, 85]	4[46, 80, 84, 85]	4[46, 70, 80, 85]	3[46, 80, 85]	2[80, 85]	1[80]	1[85]	0
	**Export and import/international trade statistics (1)**[46]	1[46]	1[46]	1[46]	1[46]	1[46]	1[46]	0	0	0	0
	**Survey (retailers) (1)**[68]	1[68]	1[68]	0	0	0	0	0	0	0	0
**Data Analysis**	**Quantitative Analysis (25)**[1, 37, 41, 48, 55, 59, 60, 62, 63, 65, 66, 68, 71-78, 81-85]	22[1, 37, 41, 55, 59, 60, 62, 63, 65, 66, 68, 71, 72, 74-78, 82-85]	22[1, 37, 41, 48, 55, 59, 60, 62, 63, 65, 66, 68, 71, 73-76, 81-85]	15[1, 48, 55, 59, 62, 65, 66, 71, 73-76, 83-85]	16[1, 37, 48, 55, 59, 62, 63, 65, 71, 72, 75, 76, 78, 81, 84, 85]	15[1, 37, 48, 59, 60, 62, 66, 71, 73-76, 82, 83, 85]	6[37, 59, 78, 81, 82, 85]	7[59, 62, 65, 73, 76, 83, 85]	2[37, 59]	2[55, 85]	1[59]
	**Flows Model (3)**[73, 76, 85]	2[76, 85]	3[73, 76, 85]	3[73, 76, 85]	2[76, 85]	3[73, 76, 85]	1[85]	3[73, 76, 85]	0	1[85]	0
	**Tax Gap (3)**[73, 76, 85]	2[76, 85]	3[73, 76, 85]	3[73, 76, 85]	2[76, 85]	3[73, 76, 85]	1[85]	3[73, 76, 85]	0	1[85]	0
	**Econometric modelling (2)**[64, 85]	2[64, 85]	1[85]	2[64, 85]	1[85]	1[85]	1[85]	1[85]	0	1[85]	0
	**Qualitative Analysis (2)**[46, 61]	2[46, 61]	2[46, 61]	1[46]	1[46]	1[46]	1[46]	0	0	0	0
	**Comparison of export and import statistics (1)**[46]	1[46]	1[46]	1[46]	1[46]	1[46]	1[46]	0	0	0	0
